# Insensitivity of Tree-Ring Growth to Temperature and Precipitation Sharpens the Puzzle of Enhanced Pre-Eruption NDVI on Mt. Etna (Italy)

**DOI:** 10.1371/journal.pone.0169297

**Published:** 2017-01-18

**Authors:** Ruedi Seiler, James W. Kirchner, Paul J. Krusic, Roberto Tognetti, Nicolas Houlié, Daniele Andronico, Sebastiano Cullotta, Markus Egli, Rosanne D'Arrigo, Paolo Cherubini

**Affiliations:** 1 Swiss Federal Institute for Forest, Snow and Landscape Research WSL, Birmensdorf, Switzerland; 2 Department of Geography, University of Zurich, Zürich, Switzerland; 3 Department of Environmental Systems Science, ETH Zurich, Zürich, Switzerland; 4 Navarino Environmental Observatory, Messinia, Greece; 5 Institutionen för Naturgeografi, Stockholm University, Sweden; 6 Dipartimento di Bioscienze e territorio, Università del Molise, Contrada Fonte Lappone, Pesche, Italy; 7 Department of Earth Sciences, ETH Zurich, Zürich, Switzerland; 8 Osservatorio Etneo, INGV, Sezione di Catania, Italy; 9 Università di Palermo, Palermo, Italy; 10 Lamont-Doherty Earth Observatory, Palisades, New York, United States of America; University of Oregon, UNITED STATES

## Abstract

On Mt. Etna (Italy), an enhanced Normalized Difference in Vegetation Index (NDVI) signature was detected in the summers of 2001 and 2002 along a distinct line where, in November 2002, a flank eruption subsequently occurred. These observations suggest that pre-eruptive volcanic activity may have enhanced photosynthesis along the future eruptive fissure. If a direct relation between NDVI and future volcanic eruptions could be established, it would provide a straightforward and low-cost method for early detection of upcoming eruptions. However, it is unclear if, or to what extent, the observed enhancement of NDVI can be attributed to volcanic activity prior to the subsequent eruption. We consequently aimed at determining whether an increase in ambient temperature or additional water availability owing to the rise of magma and degassing of water vapour prior to the eruption could have increased photosynthesis of Mt. Etna's trees. Using dendro-climatic analyses we quantified the sensitivity of tree ring widths to temperature and precipitation at high elevation stands on Mt. Etna. Our findings suggest that tree growth at high elevation on Mt. Etna is weakly influenced by climate, and that neither an increase in water availability nor an increase in temperature induced by pre-eruptive activity is a plausible mechanism for enhanced photosynthesis before the 2002/2003 flank eruption. Our findings thus imply that other, yet unknown, factors must be sought as causes of the pre-eruption enhancement of NDVI on Mt. Etna.

## Introduction

Early detection of precursors to volcanic eruptions is important in preventing major damage and loss of life. To date, these precursors have mainly included seismic, geochemical, petrographic, ground deformation and gravimetric changes that are used to assess volcanic activity shortly before eruptions (e.g., [1–6]). Surface deformation, small earthquakes, and release of volcanic gases are typically triggered by the ascent of magma in volcanoes [7]. Volcanic monitoring by remote sensing includes the acquisition of different geochemical and geophysical parameters which record volcanic processes, such as gas emissions and hydrological variations, over time (e.g., [8–10]). Using remote sensing data, an increased Normalized Difference Vegetation Index (NDVI) was observed along subsequent eruptive fissures on Mt. Nyiragongo (Congo) and Mt. Etna (Italy), as early as two years prior to eruptions of both volcanoes [11]. NDVI is closely associated with the amount of photosynthetically active radiation intercepted by vegetation, and thus with both the spatial coverage of green biomass and the chlorophyll content in leaves [12,13]. On Mt. Etna, the enhanced NDVI signal [11] was detected along a narrow line on the northeastern flank; this line later developed into an eruptive fissure during the 2002/2003 flank eruption, suggesting that enhanced photosynthesis may be related to a coming volcanic eruption.

The observed NDVI signal raises the question of whether, and how, eruptive precursor activity could influence photosynthetic rates. A comparison of tree growth with environmental parameters is necessary to estimate their influence on tree growth and to assess the potential contribution from volcanic activity. Increased photosynthesis may be induced by a number of environmental factors that are affected by pre-eruptive volcanic processes, but the most probable are an increase in heat or water availability associated with volcanic degassing (e.g., [14–16]).

Trees in temperate climates form annual growth rings, and variations in their tree-ring characteristics (width and density) reflect changes in the environmental conditions in which they grow. At high elevations and high latitudes, where the limiting factor is summer air temperature, tree growth is typically enhanced during warm summers (e.g., [17,18]). Conversely, in semi-arid ecosystems, such as in Mediterranean lowlands, growth is primarily regulated by precipitation and is enhanced during wet years [19,20]. Consequently, tree rings, often used as indicators of photosynthetic rates [21], also serve as useful proxies for climate [22,23].

The Mediterranean region is characterized by hot and dry summers and mild, humid winters [24,25]. Maximum rainfall occurs predominantly in autumn and sometimes during winter [26]. Precipitation minima and temperature maxima coincide with the period of most intense solar radiation, limiting water availability during the summer season (e.g., [27]). High rainfall variability over the year greatly affects drought severity and hampers growth [28]. Rainfall during spring is the most important factor influencing tree growth and vegetation activity of Mediterranen forests, particularly at more xeric, low-elevation sites, as also shown by remote-sensing-based model simulations and tree-ring-based growth analyses [29]. In more temperate high-elevation conditions, drought often has a minor impact on tree growth because precipitation is less limiting. In the high-elevation forests on Mt. Etna where the increased NDVI prior to the eruption was detected [11], tree growth might be enhanced by increased air temperature during the vegetation period or, given the southern latitude, by increased water availability.

Here we analyse the relationships between ring-width indices of *Pinus nigra* J.F. Arnold and monthly precipitation and air temperature, and compare our ring-width series from Mt. Etna with series from trees growing at similar elevations in Calabria, a region located at a similar latitude on the Italian peninsula without the direct influence of volcanic activity. Our hypotheses are that i) ascending magma led to an increase in local ambient air and soil temperature which positively influenced photosynthesis rates and tree growth, and that ii) water vapour from volcanic degassing locally provided additional humidity/moisture/water which became available to trees influencing photosynthesis rates and tree growth. To address these issues we assess to what extent tree-ring growth at the highest elevations on Mt. Etna is influenced by climate, i.e. air temperature and precipitation, to indirectly determine i) whether an increase in temperature caused by an incipient volcanic eruption (e.g., [30,14]) would likely induce higher photosynthetic productivity, and ii) whether, at specific locations close to rift zones, additional water availability induced by degassing of water vapour associated with the rise of magma prior to an eruption (water is the most abundant component in volcanic gas, [16]) would likely increase the photosynthetic capacity of Mt. Etna's trees (see [11]).

## Materials and Methods

### Study area

Mt. Etna is a stratovolcano situated in the northeastern part of Sicily. With an area of approximately 1600 km^2^ and a summit elevation of roughly 3330 m a.s.l., Mt. Etna is an isolated high mountain exposed to air masses from the Mediterranean Sea. The climate on Mt. Etna is strongly maritime on the eastern flank [31,32], with drier conditions on its western flank [33]. The slopes are characterized by lava flows of different ages [34]. Most of the lower elevations, being especially fertile, have been settled and used for agriculture for thousands of years. The higher elevations, from 1000–1600 m a.s.l., are dominated by European beech (*Fagus sylvatica* L.), and from 1600 m to treeline (~2000 m a.s.l.) by Corsican black pine (*P*. *nigra*). Though the treeline climatically determined at such latitudes would otherwise be higher [35,36], eruption-induced wildfires and the lack of soils on lava flows hinder its uphill development [37–39].

Besides volcanic eruptions and lava flows on Mt. Etna, other volcanic processes, such as degassing through small vents, are also present but difficult to quantify [40]. The soils of Mt. Etna are primarily classified as Regosols, Eutric or Dystric Cambisols and (Mollic) Andosols. The characteristics of these soils predominantly depend on the surface age of the lava flow and volcanic deposits from which they have developed [38,41,42]. In general, soils at intermediate to high elevation on Mt. Etna (i.e. above 800 m a.s.l.) are mostly described as Andisols with a sandy loam texture, vitric characteristics, an udic moisture regime [39] and good water holding capacity [43]. However, less mature, young soils on fresh lava flows may be less developed resulting in lower water holding capacity.

The forests around the flanks of Mt. Etna are greatly affected by both natural and anthropogenic disturbances, such as wildfires, lava flows, avalanches and logging. At the lowest elevations, from the plains up to 900 m a.s.l., agricultural crops and orchards, e.g., orange, lemon, almond, pistachio, and chestnut, are found. Only a few forest stands, mainly at the highest elevations on the northern or northeastern side of the mountain, are undisturbed [31]. Meteorological station data from Linguaglossa (530 m a.s.l., 15°08'42" E, 37°50'27" N, timespan: 1893–2004) based on daily temperatures and precipitation measurements give an average annual temperature of 18°C and a total annual precipitation of 1400 mm. Additionally, monthly temperature averages in winter are above zero at at all stations.

### Sampling

**Fig 1:**
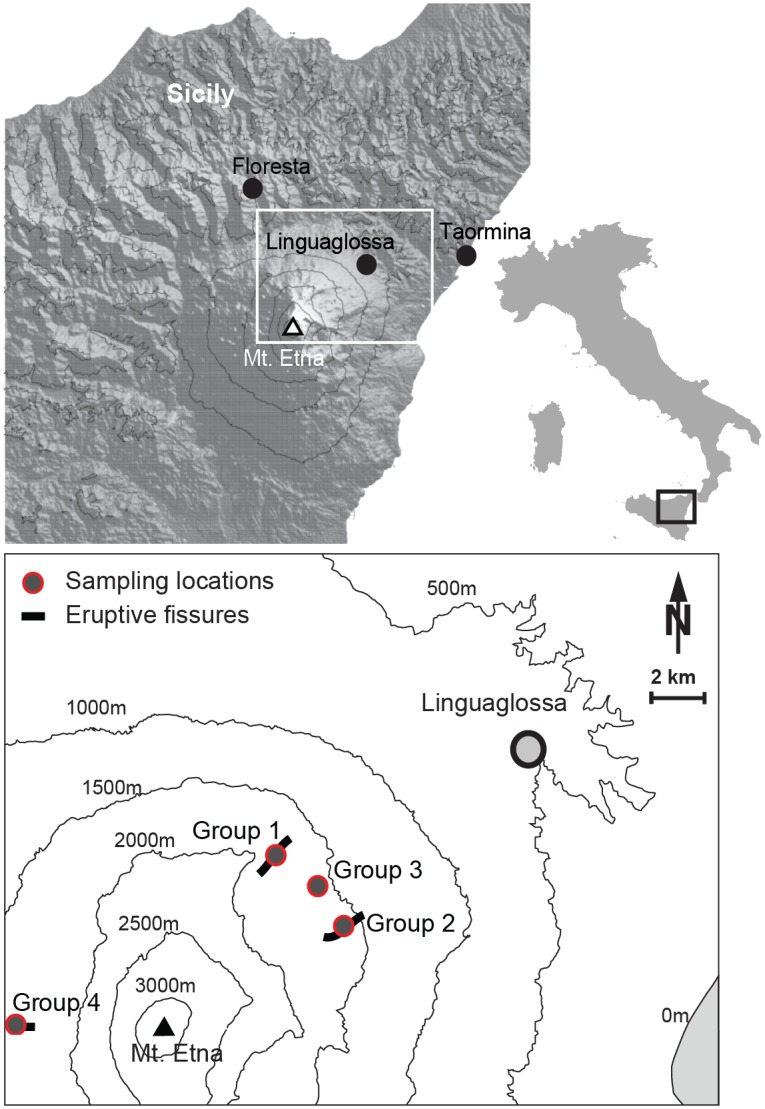
Map of sample locations. Mt. Etna sample sites (Group 1–4) on the northeastern and western slopes at an elevation range from 1600 to 1850 m a.s.l. indicating the location where samples were taken and the location of the meteorological stations. The NDVI anomaly overlays with the 2002 fissure line. A more detailed map can be found in Houlié et al. (2006). The map of Italy was created using the program R (Version 3.1.3; URL: http://www.R-project.org/) [45], the topographic map showing Sicily was created using Generic Mapping Tools (Version 5.2.1; URL: http://gmt.soest.hawaii.edu/) [46] and the basis map was taken from Egli et al. (2007) [47].

In total, we sampled 143 trees (*P*. *nigra*), with permission issued by the local forest authorities (Corpo Forestale della Regione Siciliana, Distaccamento di Bronte, Piazza Cadorna 11, I-95034 Bronte, Catania, Italy), at four high-elevation (1500 to 1900 m a.s.l.) forest sites on the northeastern and western slopes of Mt. Etna (Fig 1): 52 trees growing close to the 2002/2003 eruptive fissure (Group 1), 27 trees growing close to the 1928 eruptive fissure (Group 2), 38 trees growing in the same elevation band but far from any obvious fissures (Group 3), and 26 trees growing close to the 1974 eruptive fissure (Group 4). All sites are dominated by *P*.*nigra* and *Fagus silvatica* and are located at a comparable elevation and slope with NNE aspect except for group 4, which is located on the western flank on a western-aspect slope. Apart from that, there were no evident differences between the four sites in terms of forest-stand density, composition of tree species, topography and slope (about 14%). From each tree, two 0.5 cm diameter cores were taken orthogonally with respect to each other using a corer with a three-threaded auger by Haglof (Haglof Inc., Sweden), wrapped in paper and transported to the laboratory. All samples were mounted on wooden supports and cut using a microtome at an angle of roughly 30° to the radial axis of the tree to prevent core breakage [44]. For later comparisons, three ring-width chronologies, located close to Mt. Etna, derived from coniferous trees uninfluenced by Mt. Etna growing at similarly high elevations in Calabria (Gambarie, Monte Pollino and Sierra da Crispo) were retrieved from the International Tree-Ring Databank (ITRDB, NOAA, U.S.A.)

### Ring-width measurements

All ring widths were measured to the nearest 0.01 mm using a Leica Wild M32 binocular microscope (Leica, Germany) with 25-50x magnification, coupled to a LINTAB measuring table and computer with TSAPwin (Time Series Analysis Program) software (RinnTech, Heidelberg, Germany). Core measurements were visually crossdated against each other and any inconsistencies, if found, were eliminated. Subsequent crossdating of the single-tree chronologies with their respective mean site chronology by visual and statistical measures was performed using TSAPwin and COFECHA (50-year segments with a 25-year overlap) [48,49]. Since the sampling dates of all trees were known, crossdating was primarily used to ensure that prominent tree-ring patterns were not shifted between trees and no rings were missing.

### Meteorological data

We used monthly precipitation and air temperature data recorded at three meteorological stations on Mt. Etna: Floresta (1275 m a.s.l., 14°54'31" E, 37°59'15" N, Timespan: 1924–2004), Linguaglossa (530 m a.s.l., 15°08'42" E, 37°50'27" N, Timespan: 1893–2004) and Taormina (248 m a.s.l., 15°17'34" E, 37°17'34" N, Timespan: 1906–2004). Although longer records are available for Linguaglossa and Taormina, we only used data from 1924–2004 at all three sites so that they could be compared over a common period. In addition, interpolated monthly temperature, precipitation, cloud cover and Palmer Drought Severity Index (PDSI) data for the Mt. Etna region and Calabria from the Climatic Research Unit, University of East Anglia, Norwich, U.K. (CRU) [50] were compared to the above-mentioned station data and the tree-ring data [51,52]. Opposed to temperature and precipitation data, the PDSI incorporates both temperature and precipitation, representing long-term drought taking prior months' condition into account. On the other hand, delayed water runoff from snow during spring is not accounted for in the index (e.g., [53]). Correlations between the data recorded at the meteorological stations and the interpolated datasets were calculated, to assess whether the interpolated data could be used to further analyse relationships between climate and tree growth.

### Data analysis

All raw measurement series were standardized using the program ARSTAN (http://www.ldeo.columbia.edu/tree-ring-laboratory) by applying 30-year spline detrending combined with a variance stabilization, to remove the age trend and produce detrended ring-width indices [48,54]. All chronologies were used individually to analyse the relationships between climate and growth at different sampling sites.

We performed correlation analysis and response function modelling to quantify the influence of climate on tree growth [55]. We tested the statistical significance of temperature, precipitation, cloud cover and Palmer Drought Severity Index (PDSI; e.g., [56,57]) in different months and seasonal combinations of monthly values, including prior-year values, using Spearman rank correlation. Linear regression, as described by [58], was used to remove long-term trends in the meteorological data and avoid artificially inflating correlation values. Based on results from simple Spearman rank correlations between all the Mt. Etna chronologies and monthly meteorological data, we built "Visual Regression Models" (VRM) which were defined as standard multiple linear regression models including statistically significant (*p* < 0.05) monthly variables or monthly groupings.

In addition to VRM, Stepwise Linear Regression Modelling (SLRM), based on the Akaike Information Criterion (AIC) and using a forward-backward approach [59], was used to identify those climate variables that explained the greatest variance in each chronology on Mt. Etna. The SLRMs were based on monthly variables and groupings that defined the spring and summer seasons. For all models we only used variables that did not overlap in time (e.g., precipitation in May and precipitation in spring would not be used together because both contain May precipitation values).

The models (SLRM) were tested for collinearity among explanatory variables using variance inflation factor (VIF) analysis [60]. VIF values were all lower than 4, implying a lack of strong collinearity among explanatory variables [61]. This led to selecting the final climate models based on the AIC.

We compared how well the two model types (VRM and SLRM) explained the climate—ring width relationship:

1. Qualitative differences between the two model types included differences in monthly parameters used in the models.
2. Quantitative differences, comparing adjusted-R^2^, show the increase/decrease of explanatory power from one model type to another.

To test the reliability of the models, as well as the degree of overfitting due to including too many variables during the stepwise model selection process, we divided the timespan covered by both meteorological and ring-width measurements at Mt. Etna (1924–2004) and Calabria (1924–1980) into two segments [62,63]. The models were run on both segments to compare differences over time. In addition, the two segments were used for both forward- and backward validation to quantify model robustness over time [53].

On Mt. Etna, forward validation used a model based on the time segment 1925–1964 to predict tree growth during 1965–2004, and backward validation used a model based on the latter time segment to reconstruct 1925–1964 tree growth. For the Calabria chronologies we used the same validation procedure based on the two time segments from 1925–1951 and 1952–1980. AIC was used to test for overfitting.

We calculated how the explanatory power (R^2^) varied through time by applying our SLRMs to a 15-year moving window with 14 years overlap to identify periods of higher and lower correlations between ring-width and our climate models [52].

## Results

**Fig 2:**
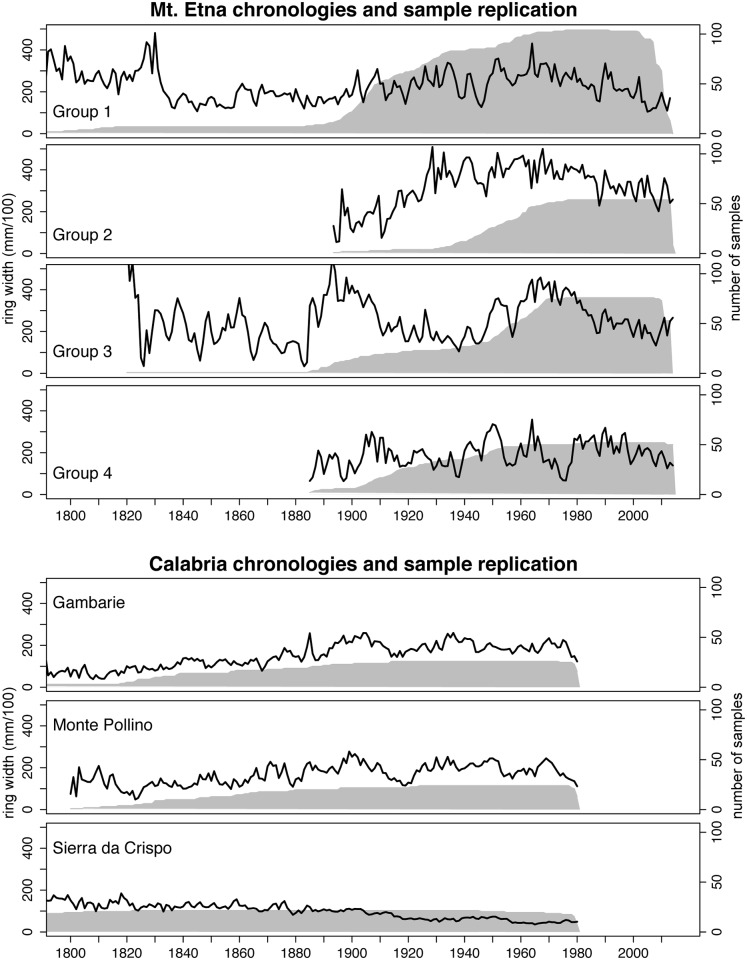
Chronologies and sample replication. Average chronologies (black lines) and sample replication of all samples (grey area) of Mt. Etna (Group 1–4) and Calabria (Gambarie, Monte Pollino and Sierra da Crispo.

The Mt. Etna chronologies show higher variability in their ring-width patterns than the chronologies from Calabria (Fig 2). The four raw ring-width chronologies (Group1-4) from Mt. Etna display growth patterns that are strongly influenced by the establishment of new generations of trees. Young trees, most germinating after 1950, greatly increase the mean ring-width, producing a clear age trend (Fig 2). Tree age ranges from 55 to 122 years on Mt. Etna, and from 128 to 299 years in Calabria. Descriptive chronology statistics are given in Table 1.

**Table 1:** Sample overview information. Descriptive statistics of sample chronologies (Group 1–4) from Mt. Etna, and the chronologies from Calabria (Gambarie, Monte Pollino and Sierra da Crispo) displaying number of series (core-series), total length (years) of group chronologies, series intercorrelation (measure of common growth signal in the chronology), mean sample length (years), elevation (m a.s.l.) and species.

		no. of series	total length	ser.interc.	mean length	elevation	species
**Mt. Etna**	Group 1	104	229	0.498	97	1850	*Pinus nigra*
	Group 2	54	121	0.577	67.1	1700	*Pinus nigra*
	Group 3	76	195	0.529	82.7	1600	*Pinus nigra*
	Group 4	52	130	0.597	95	1670	*Pinus nigra*
**Calabria**	Gambarie	26	191	0.344	130.7	1850	*Abies alba*
	Monte Pollino	24	181	0.403	128.2	1720	*Ablies alba*
	Sierra da Crispo	22	540	0.421	299.1	2000	*Pinus leucodermis*

The monthly meteorological data show stronger inter-station correlations for air temperature (Spearman correlation coefficients up to r = 0.86; *p* < 0.01) than for precipitation (up to r = 0.83; *p* < 0.01), as usually reported in the literature (e.g., [51,64]). In addition, we find that instrumental station data also correlates significantly with the interpolated CRU data (highest Spearman r = 0.67 to 0.87 for temperature; r = 0.67 to 0.78 for precipitation; *p* < 0.01). Given these correlations, comparable to those found in previous studies [51], we used the interpolated data to assess the climate influence on tree growth.

In general, total summer precipitation produces similarly high correlation values with tree growth as single monthly variables in the same season. Average spring and summer temperature and total spring precipitation produce generally lower correlations than single months during the same season (Table 2). Prior-year precipitation and temperature were also considered but results are not reported because the current year's correlations are higher. Cloud coverage is not significantly correlated with tree growth. In contrast to all other sites, tree growth at Group 3 exhibits a significant negative correlation with PDSI from April to December (results not shown).

**Table 2:** Climate-ring width correlation statistics. Spearman rank correlations between climate variables and detrended ring width from Mt. Etna chronologies (Group 1–4) and from Calabria chronologies (Gambarie, Monte Pollino and Sierra da Crispo), where P = precipitation, T = temperature, tot. = total amount of precipitation, avg. = average temperature, and prior = prior year. Values printed in bold are statistically significant with (* = *p* < 0.05, ** = *p* < 0.01, two-tailed). The significance threshold at Mt. Etna is lower than in Calabria (*r* = 0.222 vs. *r* = 0.271, respectively) because the climate and tree ring records overlap for longer on Mt. Etna than in Calabria (81 vs. 57 years, respectively).

	Mt. Etna				Calabria		
SPEARMAN rank correlations	Group 1	Group 2	Group 3	Group 4	Gambarie	Monte Pollino	Sierra da Crispo
T February	0.176	0.189	0.217	0.055	0.209	0.184	0.114
T March	** 0.427	* 0.265	** 0.327	0.197	0.152	0.163	0.018
T April	0.144	-0.109	0.064	0.169	0.148	** 0.490	0.203
T May	-0.106	-0.213	-0.15	-0.033	-0.018	* 0.271	0.061
T avg. spring	** 0.337	0.197	* 0.284	0.14	0.21	** 0.348	0.11
T June	0.035	-0.094	-0.004	0.172	* -0.283	-0.019	-0.068
T July	-0.158	* -0.223	-0.136	0.06	* -0.296	-0.068	0.026
T August	** -0.326	-0.182	** -0.397	-0.032	** -0.408	-0.05	-0.131
T September	-0.117	-0.147	-0.198	-0.01	-0.178	0.026	0.117
T avg. summer	-0.172	-0.206	-0.203	0.088	** -0.375	-0.07	-0.084
prior P December	-0.16	* -0.261	** -0.304	-0.028	0.087	0.181	0.111
P January	0.131	-0.095	0.091	0.021	-0.137	-0.018	0.059
prior P winter	0.027	* -0.251	-0.078	0.043	0.03	0.184	0.182
P February	-0.048	-0.113	-0.159	-0.125	-0.057	-0.128	0.029
P March	* -0.279	-0.151	-0.12	-0.079	-0.007	-0.001	0.044
P April	0.009	0.121	0.15	0.103	-0.22	* -0.279	-0.031
P May	0.185	0.088	* 0.227	0.025	0.209	-0.026	-0.179
P tot. spring	* -0.228	-0.104	-0.107	-0.095	-0.19	* -0.308	-0.015
P June	0.114	0.03	* 0.236	-0.117	* 0.279	0.138	0.076
P July	* 0.279	0.092	* 0.222	* 0.225	* 0.303	** 0.416	0.186
P August	0.165	-0.043	0.209	0.09	0.083	0.083	0.136
P tot. summer	* 0.271	0.09	** 0.346	0.118	* 0.325	* 0.314	0.24

In Calabria the Gambarie chronology is primarily sensitive to summer temperature and summer precipitation (Table 2). The Monte Pollino chronology responds more to spring temperatures as well as spring and summer precipitation, and the chronology from Sierra da Crispo is not significantly correlated with any climate variables. The Mt. Etna chronologies correlate with spring and summer temperatures (Groups 1–3), as well as spring and summer precipitation (Groups 1 and 3). Group 4 has no significant correlations with climate. Over all months and seasons the Mt. Etna (Groups 1–4) chronologies are, on average, less strongly correlated with temperature and precipitation than the Calabria chronologies are. The difference in the (absolute value) strength of the correlations between Mt. Etna and Calabria was not, however, statistically significant (Student *t*-test).

**Fig 3:**
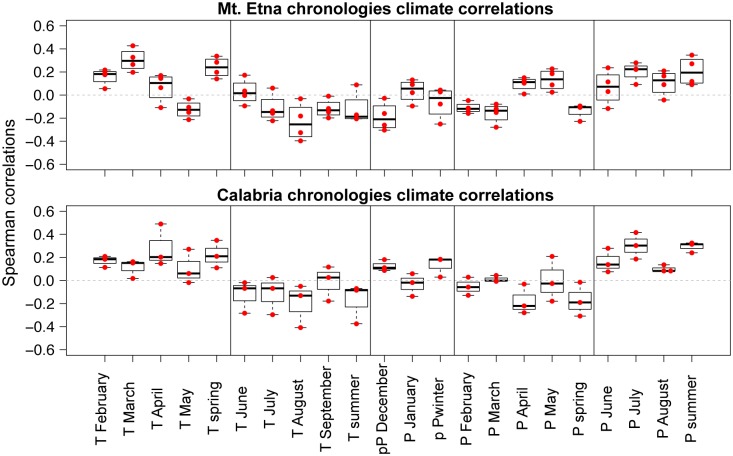
Climate—ring-width correlations. Spearman rank correlation data points of single months and seasonal groupings (red dots) of chronologies from Mt. Etna (top panel) and Calabria (bottom panel) showing the correlation range of each monthly- or seasonal variable with the different group-chronologies; where T = temperature, P = precipitation and p = prior year. Boxplots show the median and lower and upper quartiles, and the whiskers display the minimum and maximum values.

Overall we observe similar responses in the chronologies from Mt. Etna and in those from Calabria (Table 2 and Fig 3). The raw numbers of significant correlations between temperature or precipitation and ring width are broadly similar (20 out of 88, or 23% at Mt. Etna, and 14 out of 66, or 21%, in Calabria).

We used the sign test to evaluate whether the correlations between climate and ring width were positive or negative more often than expected by chance. If we pool both the Mt. Etna and Calabria correlations (Table 2), we see that ring widths are positively correlated with spring temperature (22 of 28 month and site combinations; *p* < 0.01 by the two-tailed sign test), negatively correlated with summer temperature (22 of 28 correlations; *p* < 0.01 by the two-tailed sign test), and positively correlated with summer precipitation (18 of 21 correlations; *p* < 0.05 by the two-tailed sign test), but not significantly correlated with spring precipitation (17 negative correlations out of 28 month and site combinations; *p* > 0.05 by the two-tailed sign test). Thus tree growth in these mountain environments tends to be favoured by warm springs (suggesting that water is not limiting in the springtime) and cool and relatively wet summers.

Comparing the VRM and SLRM modelling results (Table 3), we distinguished between models explaining ring width on Mt. Etna and models explaining ring width in Calabria. SLRM's yielded average R^2^ values of 20% (8% to 33%) on Mt. Etna and 26% (13% to 39%) in Calabria, demonstrating that precipitation and temperature are not strongly correlated with tree growth compared to other climatic regions. The regression models include both precipitation and temperature variables from spring and summer. There are, however slight differences between the models (Table 4).

**Table 3:** Statistics of climate models. Overview of model R^2^ and adjusted R^2^ statistics of the Mt. Etna and Calabria chronology models. Visual Regression Models (VRM) are shown in the left panel, Stepwise Linear Regression Models (SLRM) in the middle panel and the percentage of "model-improvement" from VRM to SLRM is shown in the right panel.

	Visual Regression Models			Stepwise Linear Regression Models			Model improvement (%)	
	R^2^	adj. R^2^	*p-value*	R^2^	adj. R^2^	*p-value*	R^2^	adj. R^2^
Group 1	0.23	0.19	*<0*.*01*	0.29	0.24	*<0*.*01*	6	5
Group 2	0.09	0.04	*0*.*13*	0.09	0.06	*0*.*03*	n/a	n/a
Group 3	0.27	0.22	*<0*.*01*	0.33	0.27	*<0*.*01*	6	5
Group 4	0	-0.01	*0*.*96*	0.08	0.06	*0*.*03*	n/a	n/a
**average Mt. Etna**	***0*.*15***	***0*.*11***	***0*.*27***	***0*.*2***	***0*.*16***	***0*.*02***	***6***	***5***
Gambarie	0.2	0.17	*<0*.*01*	0.26	0.23	*<0*.*01*	6	6
Monte Pollino	0.3	0.24	*<0*.*01*	0.39	0.34	*<0*.*01*	9	10
Sierra da Crispo	-	-	-	0.13	0.1	*0*.*02*	n/a	n/a
**average Calabria**	***0*.*25***	***0*.*2***	***<0*.*01***	***0*.*26***	***0*.*22***	***0*.*01***	***7*.*5***	***8***

**Table 4:** Model variables used by climate models. Climate variables (single months and seasonal groupings) used in VRM and SLRM models. Monthly variables included in the models are designated as X. Due to time overlaps between single months and seasons, variables that were excluded from the models are designated as e.

		feb	mar	apr	avg.spring	may	jun	jul	aug	avg.summer	dec	jan	tot.winter	feb	mar	apr	tot.spring	may	jun	jul	aug	tot.summer
Group1	VRM		X						X						X					X		
	SLRM		X						X			X					X					X
Group 2	VRM		X					X			X		e									
	SLRM					X					X											
Group 3	VRM		X						X		X							X				X
	SLRM				X	X			X	e	X											X
Group 4	VRM																			X		
	SLRM						X							X								
Gambarie	VRM									X												X
	SLRM			X						X												
Monte Pollino	VRM			X		X										X				X		
	SLRM			X	e						X					X						X
Sierra da Crispo	VRM																					
	SLRM			X																		X

**Fig 4:**
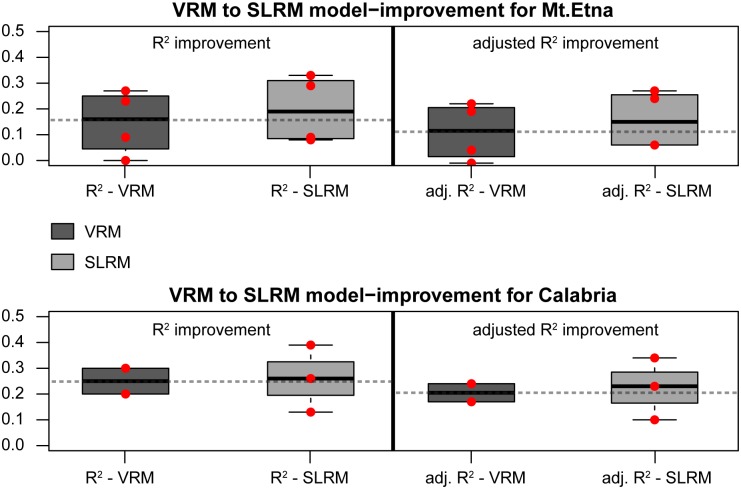
Climate model comparison. Comparisons between individual VRMs (visual regression models) and SLRMs (stepwise linear regression models) revealed only one case (Group 1) where the VRM and the SLRM were both significant (*p* < 0.05) and where a statistically significant model improvement (*p* < 0.05) was calculated. On Mt. Etna, the Group 1 model significantly improved from adjusted R^2^ = 0.19 (VRM) to adjusted R^2^ = 0.24 (SLRM). Details are summarized in Table 3.

We calculated an average improvement in adjusted R^2^ from VRM to SLRM of 5% on Mt. Etna and 8% in Calabria (Table 3; Fig 4).

When comparing VRM and SLRM which were statistically significant (*p* < 0.05), a significant model-improvement (*p* < 0.05) was only obtained with Mt. Etna's Group 1 models. These results show that on Mt. Etna even complex models such as our SLRM are not able to explain tree-growth variability much better, demonstrating that tree growth is further influenced by parameters other than climate which induce additional noise to our ring-width data.

When comparing differences over time by running the SLRMs on both time segments separately, on Mt. Etna only two out of eight model-runs on the two time segments led to significant R^2^ values (*p* < 0.05), whereas in Calabria the R^2^ in four out of six runs was significant (results not shown).

Mt. Etna model validations demonstrate that the only forward verification resulting in a significant R^2^ value (*p* < 0.01) was obtained with the Group 3 model. Forward validation of the Group 1, Group 2 and Group 4 models resulted in statistically non-significant R^2^ values. Further, backward validations show that all models calibrated on the second segment show a decrease to non-significant R^2^ values when run on the first segment. Out of eight validation runs (forward and backward) on Mt. Etna only one retained significant R^2^ values (*p* < 0.01). Including all validation runs (statistically significant and non-significant), forward validation lost 4% (from an average R^2^ of 0.18 in the first period to 0.14 in the second period) and backward validation lost 37% (from an average R^2^ of 0.48 in the second period to 0.11 in the first period) of explained ring-width variance on Mt. Etna.

The same calculations for Calabria showed that forward validation (earlier to later time-segment) lost 32% of the explained variance (average R^2^ changed from 0.53 to 0.21), while backward validation lost 11% (average R^2^ changed from 0.55 to 0.44). We calculated that of all six validation runs (forward and backward) in Calabria, only two backward validations retained significant R^2^ values (*p* < 0.05).

These results show that our models (SLRMs) of tree-ring data on Mt. Etna and in Calabria do not withstand the cross-validation test and demonstrate the importance of validating tree-ring based climate models, especially in regions such as the Mediterranean, where climatic factors are not strongly limiting tree growth.

**Fig 5:**
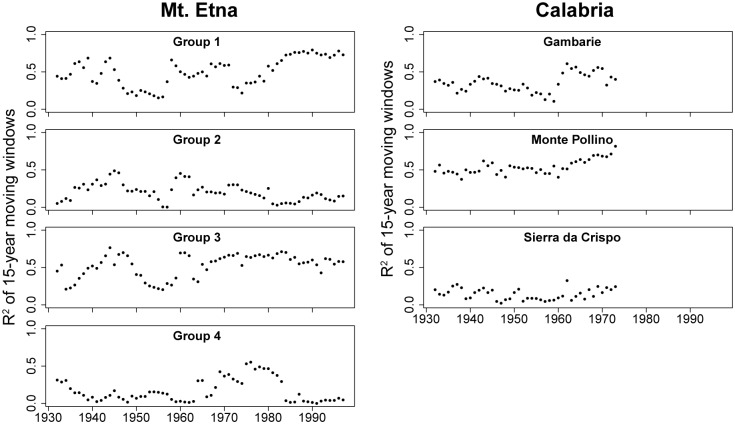
Model strength over time. Change of SLRM model goodness-of-fit statistics for 15-year moving windows over time, showing a visual suggestion of higher temporal consistency among the Calabria models.

The explanatory power of all models is generally low. The changes in the explanatory power of all models over time are shown in Fig 5. The Mt. Etna models (Groups 1–4) display a wider range of performance with some visual suggestion of a trend of increasing explanatory power over time, while the Calabria models showed less temporal variability.

## Discussion

In the Mediterranean region, seasonal drought conditions can persist at low elevations for up to five months and have a strong negative impact on tree growth [19,65], whereas at higher elevations precipitation is usually not a limiting factor [e.g., 29]. At the treeline in temperate regions, air temperature is generally the major driver of tree growth, being positively correlated with ring width (e.g., [66–70]). Similar correlations have been observed in the Mediterranean mountains as well [71,72]. At lower elevations, higher temperatures reduce tree growth by increasing evaporative demand and drought stress [73,74]. Our study showed that tree growth at high elevation on Mt. Etna is not much limited by climatic conditions: the correlations between tree-ring width and meteorological data (monthly precipitation, air temperature, PDSI and cloud coverage) were rather weak, suggesting that an increase in local moisture or temperature caused by pre-eruptive volcanic activity was unlikely to have affected tree growth. On Mt. Etna, the correlations between ring width and meteorological data were weaker than in Calabria, suggesting that the tree-ring/climate relationship on Mt. Etna might be affected by other factors. Stepwise linear regression models (SLRM) explained an average adjusted R^2^ of 16% of tree growth on Mt. Etna and 22% in Calabria. By comparison, linear regression models using spring to summer temperature at similar elevations in south-western Anatolia explained up to 51% of ring-width variance [75]. Furthermore, climate correlation values on Mt. Etna and in Calabria are lower than those found using response function analyses in the Aegean [76]. Tree-growth response to temperature across the Mediterranean region is complex and strongly affected by the change of environmental factors over longitudinal, lattitudinal and elevational gradients [77] and the age of the trees studied [78]. The dependence of tree growth on spring temperature may be reduced at sites that, like Mt. Etna and Calabria, are very close to the sea.

In general, the ring-width chronologies on Mt. Etna showed higher inter-annual variability than those from Calabria, even though the Mt. Etna chronologies were based on greater numbers of trees with a correspondingly greater averaging of random inter-tree variations. Our analyses suggest that non-climatic factors may give rise to a greater variability in tree-ring growth on Mt. Etna than in Calabria. This argument is supported by the larger differences on Mt. Etna between the regression models fitted to the two time periods.

On Mt. Etna, the correlation with summer precipitation (r = 0.27 to 0.35) is lower than in previous studies that found that water is the limiting factor in the Mediterranean region [63,75,76,79–81]. Near treeline on Mt. Etna, which is lower than at other sites at similar latitudes, because not determined by climatic conditions but rather by volcanic activities, other factors, such as higher air humidity with increasing elevation, seem to reduce the influence of summer precipitation on tree growth.

The low correlation values between PDSI and ring width on Mt. Etna (maximum r = 0.29) confirm that moisture availability is not strongly limiting tree growth. Similar results have been reported for *Pinus halepensis* Mill. in Tunisia [82] and for *Pinus sylvestris* L. in south-eastern France [83]. It is notable that *P*. *nigra* exhibits a drought-avoidance strategy characterized by efficient stomatal control of transpirational water loss [84]. At the same latitudes in Spain, the drought response of *P*. *nigra* varied along an aridity gradient with the strongest response at the most xeric sites [85]. At comparable latitudes and elevations in Calabria, studies on *F*. *sylvatica* found no correlations between either water use efficiency or basal area increment and an estimated drought index, suggesting a minimal effect of climate on tree growth during the last century [86], especially when considering the rather udic soil moisture regime at intermediate to high elevations on Mt. Etna [39]. No significant differences in soil properties, such as nitrate and ammonium or phosphorus from soils adjacent to eruptive fissures and soils away from such fissures [87] revealed homogenity of soil characteristics on Mt.Etna and demonstrated that nitrogen (nitrate and ammonium) is unlikely to have induced stronger tree growth.

Due to their high elevation and the subsequent cold, snowy, foggy and humid environmental conditions, the Mt. Etna trees do not appear to be strongly affected by Mediterranean summer drought. The combination of proximity to the sea and high elevation favours persistent seasonal fog and shading by clouds, thus limiting the vapour pressure deficit and reducing water use efficiency [88]. To survive summer droughts, vegetation uses water coming from spring precipitation or, at higher elevation, melting snow, which in some regions can be half of the annual precipitation amount [89]. At the highest elevations on Mt. Etna, winter precipitation is mainly snowfall and, based on our analyses, is not strongly correlated with tree growth (Table 2). The high porosity of the volcanic soils allows rapid infiltration, making a large fraction of the annual precipitation unavailable to the trees, and making it unlikely that winter or spring precipitation could be stored long enough to significantly alleviate summer drought stress.

Significant positive correlations between ring width and spring temperatures were found, except for Group 4. These results confirm those of previous studies on silver fir in southern Italy [52], and in southwestern Anatolia [90]. Based on the positive correlations between March temperatures and ring width on Mt. Etna, high spring temperatures appear to promote an early start of the growing season. Based on the above zero average temperatures during winter measured at all meteorological stations, possible heat discharge from the volcanic fissure in the years before the eruption (e.g., [91]) could not have caused such an early start of the growing season. In contrast, pines are able to photosynthesize during winter; thus mild temperatures in late winter may enhance the availability of reserves (non-structural carbohydrates) for allocation to cambial growth in spring. Mild conditions in spring may stimulate cambial dynamics or induce early cambial reactivation, increasing production of early-wood.

We found negative correlations between ring width and summer temperature, as previously described in Turkey [51,76,92,93], as well as in north-eastern Greece and the Spanish Pyrenees [94]. These correlations may be related to heat waves and the negative effect of drought stress on tree growth. An increase in frequency in hot and dry summers under climate change during the recent past [95] may also increase drought stress indirectly in autumn and further constrain tree growth.

Low climate sensitivity of the studied trees suggests that other factors must have caused the increased pre-eruption NDVI signal. One of the factors which could have induced fertilization is an increased carbon dioxide (CO_2_) concentration, which is commonly found in the surrounding of volcanic fissures (e.g., [96,97]).

Pre-eruption CO_2_-degassing has been observed on Mt. Etna [98] supporting the possible effects on trees analysed in this study. Even though it has been shown that the deposition of volcanic trace elements in tree rings appears to be unrelated to the occurrence of volcanic events [99] trees have been found to grow faster upon elevated concentration of CO_2_ gas in the surrounding atmosphere (e.g., [100,101]), as may be induced by emissions from the volcanic system. Analyses of tree rings have shown that trees close to natural CO_2_ springs did take up fossil CO_2_ but did not grow faster [102–104]. Depending on the amount of increased CO_2_ concentration the effects on tree growth can either be positive [105], negative [106] or even lethal, such as at Mammoth Mountain in California [107]. However, a number of studies have shown that adult, mature trees do not grow faster under elevated CO_2_ concentrations [102,108,109]. Therefore, it is not clear if an enhanced CO_2_ concentration would be a suitable candidate to explain the increased NDVI.

## Conclusions

We conclude that tree growth at the highest elevations on Mt. Etna is not significantly limited by climate. Our samples were taken near Mt Etna's upper treeline, but this treeline is not climatically determined; instead it is defined by volcanic activity and related disturbances such as wildfires. Consequently, climatic influences on tree growth are weaker than would be expected in trees growing at a climatically induced treeline where temperature is the limiting factor [29]. At the same time, the Mt. Etna trees are growing at an elevation that is too high to be strongly affected by summer drought as in Mediterranean lowlands [19]. The intermediate elevation between the two extremes (high elevation where temperature is limiting and low elevation where summer drought is limiting) makes it difficult to explain the tree-growth variability using meteorological data. The low sensitivity of tree growth to climate suggests that neither i) an increase of surrounding air temperature caused by heating from magma at shallow depths, nor ii) an increase in water availability induced by pre-eruptive subsurface pressures and water vapour, is likely to have enhanced photosynthesis before the 2002/2003 flank eruption. Thus to explain the NDVI signal previously observed [11], one must search for factors (volcanic or not) other than additional water or heat induced by volcanic activity.
